# Predicting affective valence using cortical hemodynamic signals

**DOI:** 10.1038/s41598-018-23747-y

**Published:** 2018-03-29

**Authors:** Lucas R. Trambaiolli, Claudinei E. Biazoli, André M. Cravo, João R. Sato

**Affiliations:** CMCC - Center for Mathematics, Computing and Cognition, Federal University of ABC (UFABC), Rua Santa Adélia, 166, Santo André, SP 09210-170 Brazil

## Abstract

Ascribing affective valence to stimuli or mental states is a fundamental property of human experiences. Recent neuroimaging meta-analyses favor the workspace hypothesis for the neural underpinning of valence, in which both positive and negative values are encoded by overlapping networks but are associated with different patterns of activity. In the present study, we further explored this framework using functional near-infrared spectroscopy (fNIRS) in conjunction with multivariate analyses. We monitored the fronto-temporal and occipital hemodynamic activity of 49 participants during the viewing of affective images (passive condition) and during the imagination of affectively loaded states (active condition). Multivariate decoding techniques were applied to determine whether affective valence is encoded in the cortical areas assessed. Prediction accuracies of 89.90 ± 13.84% and 85.41 ± 14.43% were observed for positive versus neutral comparisons, and of 91.53 ± 13.04% and 81.54 ± 16.05% for negative versus neutral comparisons (passive/active conditions, respectively). Our results are consistent with previous studies using other neuroimaging modalities that support the affective workspace hypothesis and the notion that valence is instantiated by the same network, regardless of whether the affective experience is passively or actively elicited.

## Introduction

Emerging theoretical models in affective neuroscience have emphasized the role of distributed patterns of brain activation in instantiating affective experiences^1,2^. Though still largely controversial^3^, recent evidence favors such network-based approaches to both mental states in general and affective phenomena in particular^4,5^. In the constructionist model, for instance, affective valence (i.e., the ability to experience pleasant/positive or unpleasant/negative sensations) is considered a fundamental and universal property of human experience^5^. In contrast, the extent to which arousal (defined as the level of excitation, which can range from low to high at any given moment) constitutes an independent dimension of affective experience remains debatable^5^. Although some researchers have suggested that subjective arousal is a fundamental property of affective experiences, its conceptualization is indeed harder and less agreed upon than that of affective valence^4,5^. Furthermore, some studies have indicated that arousal and valence are at least partially overlapping measures^6^.

Considering the critical role of valence encoding in the current biological models of affects, Lindquist and colleagues^5^ have proposed three main hypotheses as the theoretical background for their meta-analyses: (1) The bipolar hypothesis proposes that positive and negative valences represent opposite ends of a single dimension and are therefore instantiated by the same set of brain regions, implying that activity within these regions is directly related to affective value^7^; (2) the bivalent hypothesis proposes that positive and negative valences represent two distinct dimensions and are consequently evaluated by distinct brain regions^8,9^; and (3) the affective workspace hypothesis^1,10^ proposes that activity patterns of the same core neural network implement both positive and negative valences, and that valences are determined by differences in the pattern of activation. Both meta-analyses performed by this group^4,5^, which compared the results of functional magnetic resonance imaging (fMRI) and positron emission tomography (PET) studies regarding affective and discrete emotional experiences, provide evidence in support of the affective workspace hypothesis. This evidence suggests that brain regions such as the dorsomedial prefrontal cortex, supplementary motor area, ventrolateral prefrontal cortex, anterior insula, amygdala, ventral striatum, and thalamus are consistently associated with affective valence.

Despite the enormous contribution of neuroimaging to reframing research on affects, few studies have utilized functional near-infrared spectroscopy (fNIRS) to examine such experiences^11^, at least from a network-based perspective. This technique uses low-energy light detectors and transmitters to measure the light absorption of endogenous chromophores, enabling researchers to evaluate changes in the concentration of oxyhemoglobin and deoxyhemoglobin at the cortical surface^12^. Importantly, fNIRS findings can be compared to blood-oxygen-level dependent (BOLD) signals obtained via fMRI, as both methodologies rely on neurovascular coupling mechanisms^13^. Moreover, concentrations of oxyhemoglobin and deoxyhemoglobin are more direct measures of metabolic and vascular changes in response to local neural activity than BOLD signals, and fNIRS exhibits better spatial and temporal resolution than EEG and fMRI, respectively. This method is also advantageous for studying affective experiences due to the portability and low cost of the required equipment^14^. Previous fNRIS studies have focused on the localization of hemodynamic activity correlated with different aspects of affective processing, placing particular emphasis on activity in the prefrontal cortex^11,14,15^ and valence lateralization^16^.

In the present study, we first aimed to determine whether fNIRS data from fronto-temporal and occipital cortical areas enable the discrimination of affective valence. However, unlike most fMRI and fNIRS studies to date, we did not utilize univariate analyses or general linear models; rather, we used multivariate classification procedures to interpret the biological significance of the hemodynamic signal^17^. As multivariate analyses and decoding techniques enable the estimation of mental states based only on measurements of brain activity, these techniques have become increasingly common in cognitive neuroscience. Relative to traditional methods, multivariate approaches are advantageous in that they allow researchers to examine the full spatial pattern of brain activity, which is measured at many locations simultaneously^17^. This feature is particularly desirable in light of recent findings suggesting that affective dimensions are instantiated by distributed patterns of brain activation^2,18,19^.

In light of the principled approach and findings of Lindquist *et al*.^5^ meta-analysis, our second aim was to use fNIRS-based decoding of affective experiences to discuss the alternative hypotheses for the neural basis of valence. High predictive performance in separating neutral from positive and negative trials and positive from negative trials would be expected if regional activity varies monotonically with valence (i.e., the bipolar hypothesis) or if two distinct networks are involved in generating such activity (i.e., the bivalent hypothesis). Evidence regarding the likelihood of the bipolar and bivalent hypotheses could be further distinguished by considering the weights assigned by the classifier for each region evaluated^8,9^. According to the bipolar hypothesis, a region that is positively associated with positive valence should be negatively associated with negative valence and vice versa^7^. However, according to the bivalent hypothesis, a region that is positively associated with positive valence should exhibit no relationship with negative valence. In contrast, in the affective workspace hypothesis, high predictive performance is expected only for distinguishing between positive/negative valences and neutral valences, while low predictive performance is expected when comparing positive and negative valences^1,10^. Thus, if the affective workspace hypothesis holds true, then the same region would be positively associated with both positive and negative valences.

Using fNIRS, Köchel *et al*.^20^ observed increased levels of oxyhemoglobin in the occipital region during affective experiences induced by viewing pictures (passive elicitation condition), as well as increased activation in the parietal region when participants imagined affective situations (active elicitation condition). In a related fMRI study, Costa *et al*.^21^ reported that actively elicited affective experiences were associated with BOLD activity in core affective workspace regions such as the medial prefrontal cortex, amygdala, and nucleus accumbens. Moreover, two recent meta-analyses of affective neuroimaging studies^4,5^ have suggested that valence attribution is implemented by the same network, regardless of whether the affective experience has been actively or passively elicited. Accordingly, we aimed to investigate the neural underpinnings of active and passive elicitation conditions using a multivariate classification procedure. If the same network underlies valence attribution under both conditions, good predictive performance should be observed when training the classifier with passive elicitation data and testing with active elicitation data, or vice versa^17^. To achieve our aims, we designed experiments in which each participant viewed a set of images from the International Affective Picture System (IAPS) catalog that elicited positive, negative, or neutral experiences (passive elicitation condition) or was asked to imagine a positive, negative, or neutral personal experience (active elicitation condition).

## Results

We first investigated whether affective experiences with different valences, elicited under active and passive conditions, could be accurately classified using cortical hemodynamic information only. The top third of Table 1 presents the results of pairwise comparisons of affective experiences (positive vs. neutral, negative vs. neutral, and positive vs. negative) for the passive and active elicitation conditions. Excellent decoding performance was observed for positive or negative affective experiences versus neutral affective experience: When passively elicited, all accuracy values were approximately 80%, while those during active elicitation reached approximately 90%. Furthermore, the decoding of positive vs. negative valence was not significant in either condition.

**Table 1:** Means and standard deviations of decoding accuracy across participants for each tested configuration.

	Comparison			Accuracy (%)		
	Class A	Class B	(Classes A+B)/2	Class A	Class B	p-value
*Intra-block leave-one-trial-out*						
Passive elicitation	Positive	Neutral	**85.41 ± 14.43**	**84.08 ± 18.25**	**86.73 ± 13.13**	**<0.001**
	Negative	Neutral	**81.53 ± 16.05**	**78.37 ± 21.92**	**84.69 ± 13.86**	**<0.001**
	Positive	Negative	**58.37 ± 15.86**	**57.14 ± 20.00**	**59.59 ± 19.36**	**0.007**
Active elicitation	Positive	Neutral	**89.80 ± 13.84**	**88.16 ± 16.29**	**91.43 ± 13.23**	**<0.001**
	Negative	Neutral	**91.53 ± 13.04**	**89.80 ± 17.85**	**93.27 ± 10.49**	**<0.001**
	Positive	Negative	45.10 ± 21.32	44.08 ± 23.09	46.12 ± 24.90	1.000
*Inter-block cross-validation*						
Passive x Active elicitation	Positive	Neutral	56.94 ± 21.77	50.20 ± 26.18	63.67 ± 24.55	0.364
	Negative	Neutral	54.69 ± 22.72	44.90 ± 29.59	64.49 ± 25.34	1.000
	Positive	Negative	50.82 ± 13.82	53.47 ± 34.73	48.16 ± 32.89	1.000
Active x Passive elicitation	Positive	Neutral	50.10 ± 16.19	42.04 ± 25.25	58.16 ± 18.56	1.000
	Negative	Neutral	55.92 ± 18.33	50.61 ± 29.75	61.22 ± 19.43	0.341
	Positive	Negative	50.41 ± 14.28	44.08 ± 34.39	56.73 ± 33.75	1.000
*Inter-block leave-one-trial-out*						
Neutral			**83.27 ± 12.19**	**84.90 ± 13.09**	**81.63 ± 14.63**	**<0.001**
Negative	Active elicitation	Passive elicitation	**72.86 ± 17.91**	**70.61 ± 24.19**	**75.10 ± 18.50**	**<0.001**
Positive			**75.71 ± 17.68**	**77.55 ± 19.85**	**73.88 ± 20.90**	**<0.001**

The first and third configurations were analyzed using the leave-one-trial-out (LOTO) method (intra-block and inter-block LOTO, respectively), while the second configuration (inter-block cross-validation) was trained using data from either the active or passive elicitation condition and tested with data from the remaining condition. Significant accuracies (p < 0.05) are highlighted in bold.

We then examined whether training the classifier in either the active or passive condition could increase the significance of valence decoding in the remaining condition. The middle third of Table 1 shows results of decoding for this analysis. Classification accuracies were not above chance for any of the tested comparisons.

We further aimed to determine whether passive or active condition could be classified based on a single experienced valence using an LOTO approach. The lower third of Table 1 presents the results of decoding analyses for the active and passive conditions according to valence. Classification accuracy was above 70% for all combinations, indicative of good decoding performance.

**Figure 1:**
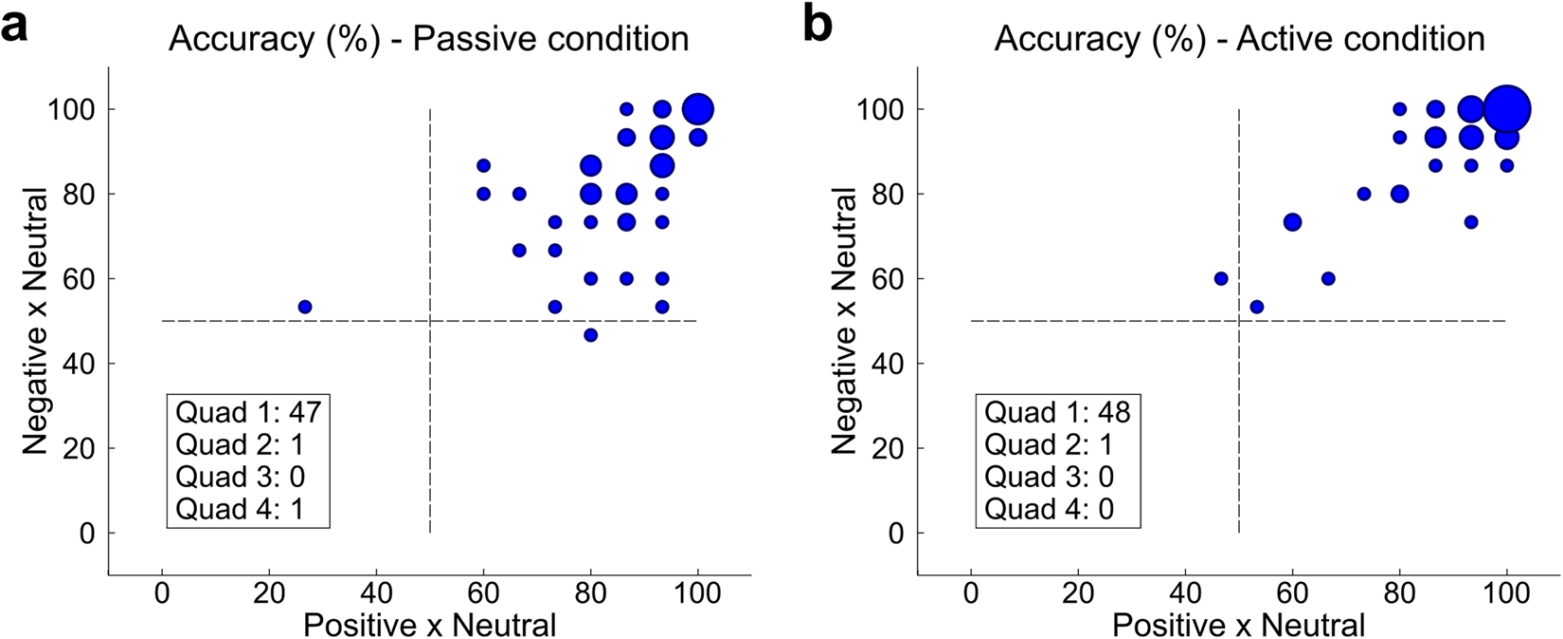
Distribution of classification accuracy values for negative vs. neutral (y-axes) and positive vs. neutral (x-axes) comparisons in the passive (**a**) and active (**b**) elicitation conditions. The diameter of the circle is proportional to the number of participants for which that level of performance was observed.

Given the high accuracy in distinguishing between positive vs. neutral and negative vs. neutral affective experiences, we further examined the classification accuracies for each participant. As shown in Fig. 1, classification accuracy for both tests and both conditions (A: passive elicitation condition, B: active elicitation condition) was above chance (50%) for virtually all participants. Moreover, for most participants, classification accuracy was above 75% (passive: 34 participants; active: 42 participants), reaching as high as 100% in both comparisons for some participants (passive: 7; active: 17).

The aforementioned results suggest that both oxyhemoglobin and deoxyhemoglobin concentration signals can be used as features for the classifier. All classification procedures were also performed separately for both signals, yielding similar results (see Supplementary Material).

**Figure 2:**
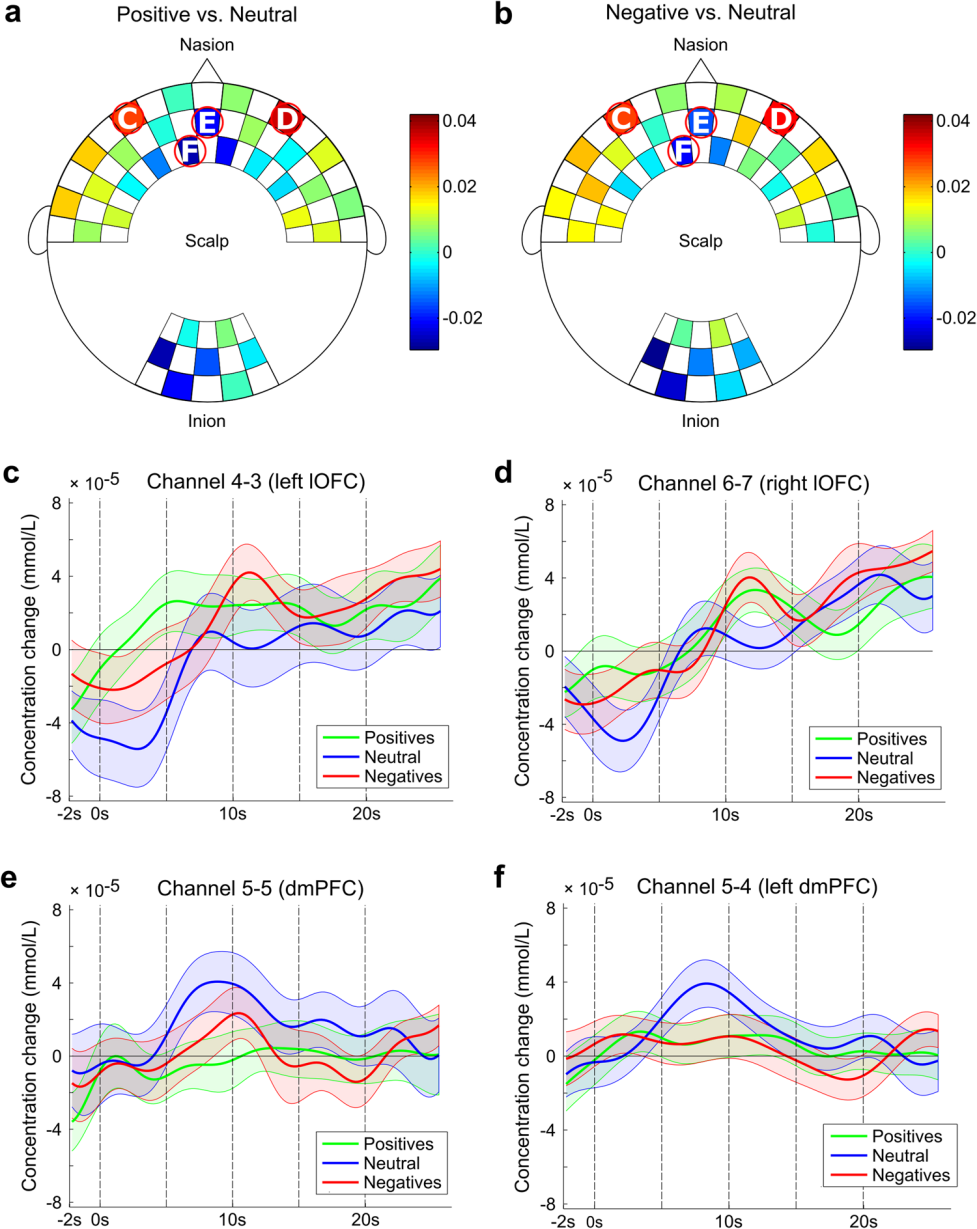
Weights assigned to the deoxyhemoglobin concentration of each channel by the linear discriminant analysis (LDA) classifier for passive elicitation decoding in positive vs. neutral (**a**) and negative vs. neutral (**b**) comparisons. Hotter colors are indicative of positive weights, while cooler colors are indicative of negative weights. Channels circled in red represent those with the highest absolute weights, while white letters indicate the corresponding subfigure that includes the block average for channels over the (**c**) left lateral orbitofrontal cortex (lOFC), (**d**) right lateral orbitofrontal cortex (lOFC), (**e**) dorsomedial prefrontal cortex (dmPFC), and (**f**) left dorsomedial prefrontal cortex (dmPFC). The continuous line represents the mean, while the shaded area represents the standard deviation, for positive (green), negative (red), and neutral (blue) affective experiences.

**Figure 3:**
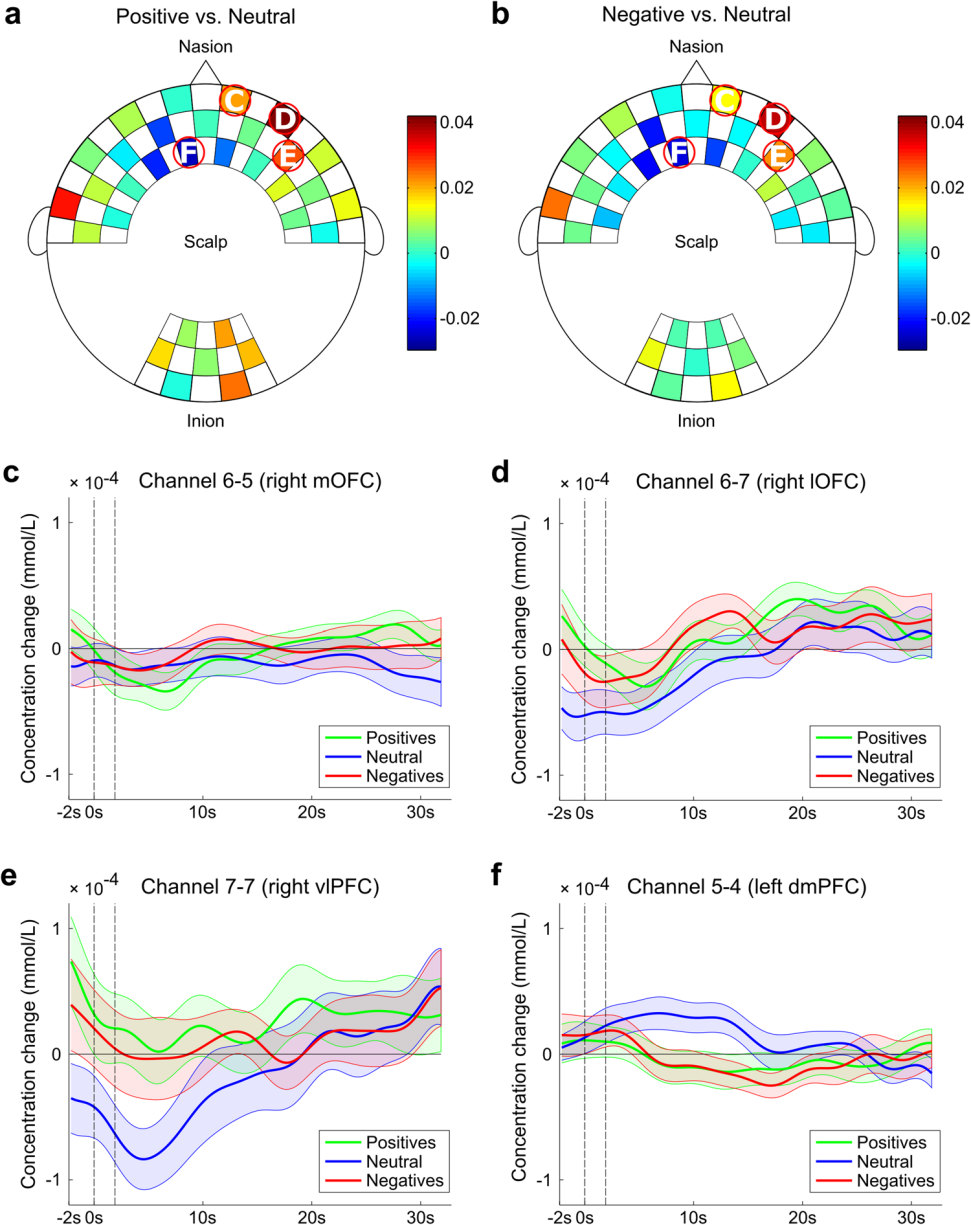
Weights assigned to the deoxyhemoglobin concentration of each channel by the linear discriminant analysis (LDA) classifier for active elicitation decoding in positive vs. neutral (**a**) and negative vs. neutral (**b**) comparisons. Hotter colors are indicative of positive weights, while cooler colors are indicative of negative weights. Channels circled in red represent those with the highest absolute weights, while white letters indicate the corresponding subfigure that includes the block average for channels over the (**c**) right medial orbitofrontal cortex (mOFC), (**d**) right lateral orbitofrontal cortex (lOFC), (**e**) right ventrolateral prefrontal cortex (vlPFC), and (**f**) left dorsomedial prefrontal cortex (dmPFC). The continuous line represents the mean, while the shaded area represents the standard deviation, for positive (green), negative (red), and neutral (blue) affective experiences.

We also examined the weights ascribed by the classifier to different channels for both pairwise comparisons in order to determine whether similar features are used to differentiate between positive vs. neutral and negative vs. neutral affective experiences. As shown in Figs 2a,b and 3a,b, deoxyhemoglobin concentration signals in critical channels were consistently assigned the highest weights across participants and comparisons (when comparing all maps no differences were observed, with exception to one channel - see Supplementary Material). Most relevant features included deoxyhemoglobin signals in channels above the fronto-central and occipital cortices yet were primarily concentrated near the midline of the cortical surface. Given these results and the relative ease with which deoxyhemoglobin concentration can be compared to BOLD signals^22^, we then investigated the time course of deoxyhemoglobin signals. A strong dissociation of activity between positive or negative and neutral experiences was observed in the most relevant channels. As shown in Figs 2c–f and 3c–f, channels associated with the lateral orbitofrontal cortex (lOFC), medial orbitofrontal cortex (mOFC), and ventrolateral prefrontal cortex (vlPFC) (Figs 2c,d and 3c–e) exhibited predominantly higher concentrations of deoxyhemoglobin during positive and negative experiences, while channels near the dorsomedial prefrontal cortex (dmPFC) (Figs 2e,f and 3f) exhibited higher deoxyhemoglobin levels during neutral experience. Moreover, occipital channels seemed to able to discriminate between active and passive elicitation conditions once higher weights (colors close to red) were assigned during the active elicitation condition and lower weights (colors close to blue) were assigned during the passive elicitation condition (with significant difference in channel 12-12 when comparing Figs 2b and 3b).

Our analysis of potential confounders revealed no significant effect of gender on the affective experience prediction. Furthermore, no significant correlations were observed between pre-to-post task mood variations (VAMS questionnaire) and predicted values. As expected, scores for the intensity of the affective experience differed significantly between positive and neutral ones (positive: 6.135 ± 1.628, neutral: 3.116 ± 1.374; p < 0.001) and between negative and neutral ones (negative: 7.098 ± 1.461; p < 0.001) for the passive condition. Similar differences were observed for the active condition (positive: 6.343 ± 1.752, negative: 6.708 ± 1.718, neutral: 0.431 ± 1.272; p < 0.001 for both), indicating that the volunteers were able to actively elicit the proper affective experiences in this particular condition.

## Discussion

In the present study, we aimed (i) to evaluate whether fNIRS data from fronto-temporal and occipital cortical areas contains sufficient information to discriminate between affective experiences; (ii) to examine possible differences regarding the neural instantiation of passively and actively experienced affect, and (iii) to discuss our findings in light of the best evidences available for the neural basis of affective valence.

First of all, it is important to emphasize that for both conditions the elicited affective experiences should comprise various dimensions, including valence, arousal and attentional variations (see further discussion below). Nevertheless, considering the crucial role of the valence dimension^1,4,5,23^, we speculate the relations between our results and current models for the neural basis of this dimension. For bivalent and bipolar hypotheses of valence, a high predictive performance when classifying positive versus negative experiences would be expected. However, comparisons of positive/negative versus neutral experiences using an LOTO configuration were associated with high predictive performance, while those between positive and negative experiences were associated with poor overall performance. Additionally, according to the bivalent hypothesis, there should be independent clusters of activation associated with positive or negative valences^8,9^. In such a case, a linear classifier must exhibit high accuracy for any of the affective experiences. In addition, given that clusters are supposed to be independent, the weights assigned to each channel should exhibit distinct patterns during the classification of positive or negative experiences versus neutral ones. However, the patterns were remarkably similar among the tests. This similarity between channels involved in positive and negative valence classification might support the bipolar hypothesis. However, as previously mentioned, the bipolar hypothesis can be described as a single coordinate axis, with the same network responding to both positive and negative valences in opposite ways^24^. If we consider the classifier weights to be distributed over this axis, neutral valence can be regarded as the zero point. Thus, if a region receives a positive weight when positive and neutral valences are compared, this region should receive a negative weight when negative and neutral valences are compared. Moreover, clusters of channels exhibited the same polarity of weights for both comparisons of positive/negative experiences versus neutrality. For example, during passive condition, negative weights were assigned to channels over the dmPFC, while positive weights were assigned to channels over the left and right lOFC for both comparisons. Similarly, the left dmPFC received negative weights, while the right mOFC, right lOFC, and right vlPFC received positive weights during active condition for both comparisons. In short, the pattern observed appears to be inconsistent with the bipolar hypothesis.

The affective workspace hypothesis suggests that both positive and negative valences recruit similar neural networks^1^. In this hypothesis, the affective processing network involves areas classically listed as “emotional centers” (e.g., the amygdala and ventral striatum), as well as areas more recently implicated in affective processing such as the lOFC, agranular insula, vlPFC, ventromedial prefrontal cortex (vmPFC), dmPFC, the subgenual and pregenual portions of the anterior cingulate cortex (ACC), supplementary motor area (SMA), and the lateral portions of the right temporal/occipital cortex^4,5,25^. The original affective workspace model also includes the thalamus, hypothalamus, and autonomic control centers in the midbrain and brainstem^1^.

Since fNIRS measures are restricted to portions of the cortical surface^14^, it is impossible to evaluate all regions implicated in the affective workspace. However, the features assigned the highest weight in the present study were close to some of the proposed core regions of the affective workspace model. Some channels were observed to be especially relevant and reproducible across different affective experiences and elicitation conditions. For instance, activity in the left dmPFC and right lOFC was highly relevant for classification in all affective experiences and experimental conditions. The lOFC is considered a heteromodal association area for both internal and external sensory representations^1^. Chikazoe *et al*.^23^ identified populations of neurons that responded to both positive and negative affective stimuli in several brain regions, including the mOFC and lOFC. Remarkably, the left dmPFC exhibited higher values of average deoxyhemoglobin concentration for neutral experience than for positive/negative experiences. This finding is in accordance with those of previous studies, which have identified the dmPFC as a core region of the default mode network and proposed a significant role for this region in the affective workspace network^1,26^.

For a linear classifier, if two classes of data exhibit different distributions for a given set of features, the classifier may be able to learn how to differentiate between these classes based on these features^27^. To evaluate whether active and passive elicitation conditions are associated with the same networks and/or patterns of neural activity, we performed two types of classification tests: inter-block cross-validation and inter-block LOTO analysis. In the inter-block cross-validation test, data from the passive condition were used to train the algorithm to identify valence, while data from the active condition were used to test the learned pattern, and vice versa. Thus, if the same network or pattern of neural activity underlies valence in both elicitation conditions, the pattern learned during training would be observed during testing, and good predictive performance is expected. However, trained classifiers were not effective in using information from one elicitation condition to discriminate valence based on data from the other elicitation condition, suggesting that different networks or patterns of activity are involved in passive and active elicitation.

To confirm this result, the inter-block LOTO test was used to determine whether the classifier could distinguish between active and passive conditions for each valence class. For these analyses, the classifier was trained with data from both conditions (active and passive) and tested using the LOTO approach. If both active and passive conditions are associated with the same neural network or pattern of neural activity, the classifier should not be able to correctly classify the trial that has been left out. However, high and significant accuracy values were observed for all comparisons, supporting the notion that different networks are involved in active and passive elicitation.

To determine whether the difference between active and passive conditions was associated with different neural networks or different levels of hemodynamic activation, we next focused our analysis on the weights assigned by the classifier in the intra-block LOTO comparisons of negative/positive experiences with neutral ones. Among the channels with the highest absolute weight assigned by the classifier, the channel over the dmPFC exhibited lower weights during both conditions. In contrast, the left lOFC exhibited higher weights during passive elicitation, while the right mOFC and right vlPFC exhibited higher weights during active elicitation, relative to the opposing condition.

Channels positioned over the right mOFC region exhibited positive weight values, with deoxyhemoglobin concentration for these regions exhibiting higher average values for affective experiences than for neutral experience. The mOFC was proposed to guide internal responses to affective contexts^1^. In addition, the right vlPFC exhibited higher concentrations of deoxyhemoglobin during positive and negative experiences than during neutral. The similarity of deoxyhemoglobin concentration curves for the vlPFC supports the notion that similar neural processes are involved in generating positive and negative experiences. As previously mentioned, data from the left lOFC area corresponded to the contralateral readings from the right lOFC in passive condition. Finally, passive elicitation was also associated with greater involvement of the dmPFC region during neutral trials, in accordance with its purported roles in the functional architecture of the default mode network and as a core region in the affective workspace^4,28^.

We further observed opposite weights for channels located over the occipital regions, which may have included the primary visual and unimodal association areas^29^, during passive and active conditions. During passive elicitation, these channels received mainly negative weights, while weights were either positive or approached zero during active condition. Unlike in the passive condition, participants did not respond to any stimuli in neutral experience. Thus, increases in deoxyhemoglobin concentration may have been associated with activation of visual association areas during imagination. Previous studies have demonstrated that the involvement of the occipital cortex is crucial to the quality of the active elicitation because it regulates subjective vividness during imagination^20,30^. Alternatively, higher cognitive demands involved in active condition relative to those involved in neutral experience may explain differences in the observed pattern of occipital activation among conditions.

The present study possesses some limitations of note. Although arousal is a primary component of the circumplex model of affect^31^, we did not discuss its role in relation to our findings for several reasons. First, it is difficult to properly conceptualize arousal, which can refer to the intensity of a feeling, attention, behavioral engagement, or physiological activation^5^. In addition, some studies have suggested that valence and arousal are not fully separable dimensions, but represent a single V-shaped dimension^32^. This conceptual caveat is also experimentally founded, since it is not possible to separate the two aspects in most affective stimuli systems (e.g., IAPS)^6^, as valence is accompanied by arousal changes and vice versa^1^. Self-report ratings of arousal also exhibit inter-subject variation, and some participants are unable to separate arousal from valence^33^. However, studies of emotional memory that have examined the neurophysiology of these dimensions have suggested that different pathways are involved in the processing of arousal and valence. The arousal pathway is purported to involve the amygdala and hippocampus, while the valence network is purported to include the PFC and hippocampus^34^. Thus, considering the restriction of fNIRS recording to cortical layers^12^, although both dimensions are closely linked, our decoding experiment evaluated only structures involved in the physiology of valence.

In the present study, we observed higher absolute weight values for deoxyhemoglobin concentration under all comparisons, while oxyhemoglobin showed similar values for almost all channels. The concentration of deoxyhemoglobin is thought to be more closely connected to BOLD signals than oxyhemoglobin signals^32^. Although the oxyhemoglobin concentration was not relevant to our application, it does provide complementary information to fMRI data^35^. However, fNIRS data is particularly suitable for decoding approaches, as it allows for more straightforward data analysis than EEG or fMRI^36,37^. Thus, in addition to the relative ease of fNIRS data acquisition^14^, this method is particularly advantageous due to its portability, simplicity, and computational economy, opening doors for robust applications using neurofeedback^38,39^ or naturalistic experimentation^40^. In this context, the present findings may expand the potential clinical applications of fNIRS in the treatment of affective disorders^39,41^.

However, it remains to be determined whether fNIRS is influenced by peripheral responses to affective stimuli, such changes in heart rate, blood pressure, and the aerobic process of energy consumption associated with muscle contraction^14^. Further study of the issue is required and should involve multimodal psychophysiological monitoring in conjunction with the collection of neuroimaging data.

In conclusion, our results show high classification of affective experiences, mainly when differentiating positive or negative affect and neutral ones in both experimental conditions (passive and active elicitation). Also, the patterns observed here are in agreement with current models of affect. Finally, our results suggest that fNIRS is a suitable tool for decoding mental states, bringing a promising approach for the development of affective neurofeedback systems.

## Methods

### Participants

Forty-nine healthy volunteers (25 women, 24 men; mean age ± standard deviation: 24.65 ± 3.23 years; age range: 20–34 years) provided informed consent for both study participation, and publication of identifying images. All participants had normal or corrected-to-normal vision and had no previous or current diagnosis of neurological (ICD-10: G00-G99) and/or psychiatric disorders (ICD-10: F00-F99). The experimental protocol was approved by the Research Ethics Committee of the Federal University of ABC, and all methods were performed in accordance with the relevant guidelines and regulations. No payment was provided, in accordance with local regulations.

### Experimental configuration

Participants sat in a padded chair with armrests, which was positioned 1 m away from a monitor. They were asked to remain relaxed, to keep their hands within sight (i.e., resting on the table or on the armrests), and to avoid both eye movements and bodily movements. The recording room remained dark during registration, and participants wore earplugs to minimize the influence of unrelated visual/auditory stimuli.

Each participant provided ratings of his or her mood using an 11-point Likert scale (ranging from 0 to 10) immediately before and after the experiment. Ratings were provided for the following dimensions based on the visual analogue mood scale (VAMS)^42^, which exhibits high reliability and validity across various populations^43–45^: agitation, strength, confusion, agility, apathy, satisfaction, worry, perspicacity (velocity of reasoning and clarity of ideas), stress, attention, capacity, happiness, hostility, interest, and retraction.

#### Passive elicitation condition

For passive elicitation condition, we used images available on the IAPS catalog^46^. First, all IAPS images were ranked according to their valence values. We then selected the 30 images with the highest average valence values for the positive affective experience, the 30 images with the lowest average valence values for the negative affective experience, and 60 images with intermediate values for the neutral affective experience (Table 2). Although we controlled the average values of subjectively measured arousal, neutral pictures were associated with lower arousal due to the V-shaped relationship between arousal and valence^6^. Thus, arousal values were accompanied by valence changes (and vice versa)^1^.

**Table 2:** Selected IAPS images and mean ± standard deviation values of valence and arousal.

Affective experience	Pictures	Valence	Arousal
Positive	1811, 2057, 2080, 2209, 5210, 5830, 7200, 2040, 2058, 2091, 2340, 5700, 5833, 7330, 1440, 2045, 2070, 2150, 2347, 5825, 5910, 7502, 1710, 2050, 2071, 2165, 2550, 5829, 5982, 8420	7.884 ± 0.220	5.036 ± 0.448
Negative	2375.1, 3101, 3261, 9181, 9322, 9560, 2703, 3180, 3301, 9185, 9326, 9571, 2095, 2800, 3191, 3350, 9253, 9332, 2205, 3016, 3225, 9040, 9300, 9421, 2345.1, 3062, 3230, 9140, 9301, 9433	2.007 ± 0.183	5.549 ± 0.339
Neutral	2122, 2514, 5520, 7019, 7182, 7550, 2191, 2635, 5531, 7021, 7207, 7632, 2211, 2702, 5532, 7043, 7242, 7830, 1122, 2308, 2745.1, 5533, 7052, 7248, 8065, 1350, 2377, 2850, 5740, 7053, 7249, 1616, 2381, 2870, 5920, 7058, 7365, 1675, 2385, 2880, 6910, 7062, 7497, 1820, 2487, 5395, 7001, 7080, 7500, 1908, 2495, 5471, 7014, 7090, 7506, 2102, 2499, 5510, 7017, 7100	5.234 ± 0.060	3.770 ± 0.813

Each block consisted of 20 trials (five for positive experience, five for negative experience, and 10 for neutral experience). For the first 2 s of each trial, a white cross was presented in the center of a blank screen. Over the next 30 s, a new image was presented every 5 s, resulting in a total of six figures per trial for each affective experience. At the end of each trial, a new screen was presented, and the participant was asked to rate the intensity of the affective experience during the trial on a scale ranging from 1–9 (1: no affective experience; 9: highly intense affective experience). A blank screen was then presented for a random duration between 2–4 s. Participants were instructed to blink during this period but not during the other phases of the experiment.

Trial order was randomized across participants, with a neutral trial between negative and positive trials. To provide examples of negative and positive affective experiences for further imagery, the passive elicitation block was the first block for all participants.

#### Active elicitation condition

**Figure 4:**
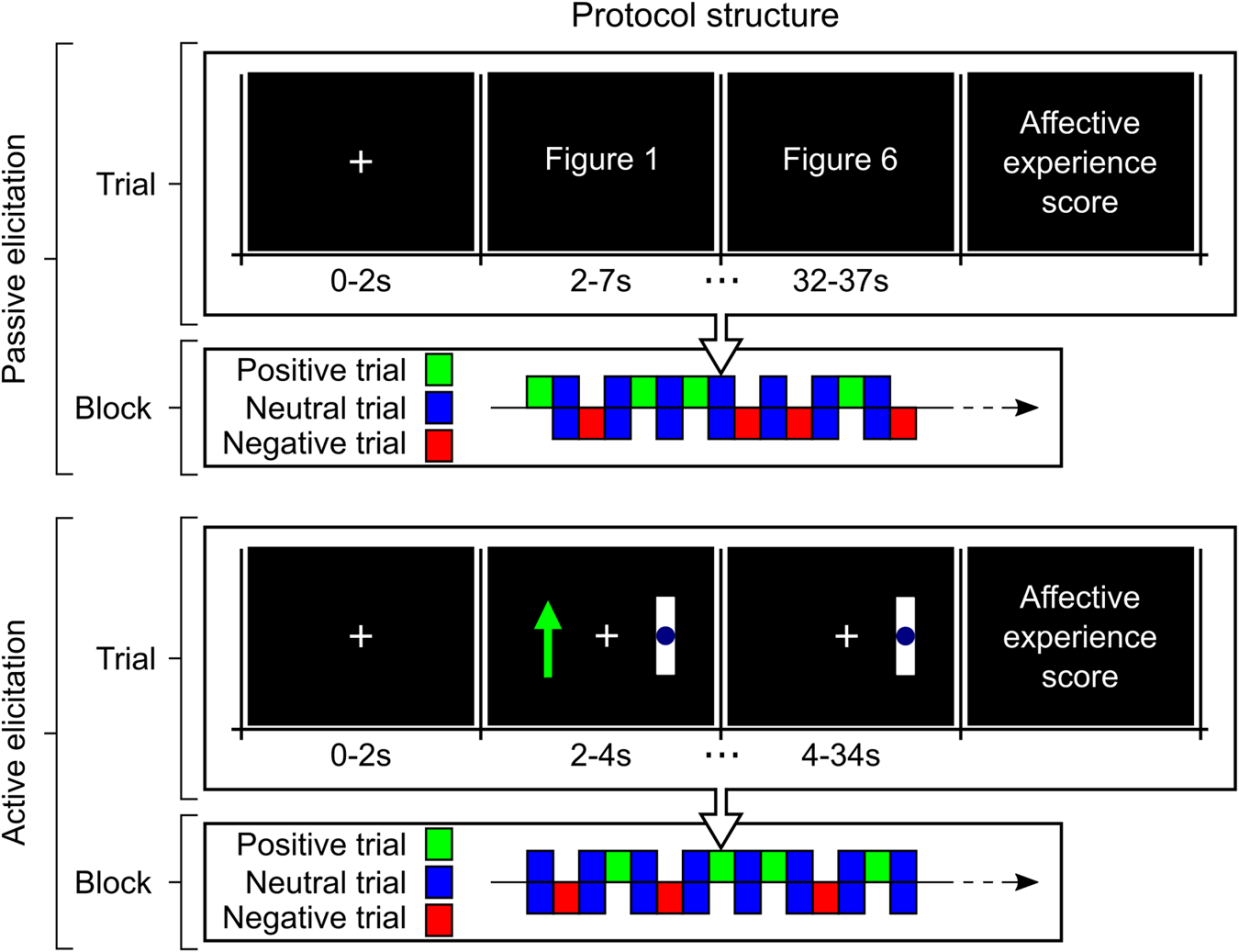
Experimental design. Visual stimuli in passive elicitation condition (0–2 s: baseline; 2–37 s: target recording). Visual stimuli in active elicitation condition (0–2 s: baseline; 2–4 s: instruction; 4–34 s: target recording). Trial order was randomized across participants, with a neutral affective experience between negative and positive affective experiences.

The active elicitation condition consisted of 20 trials (five for positive affective experience, five for negative affective experience, and 10 in which the participant rested with his or her eyes open). For the first 2 s of each trial, a white cross was presented in the center of a blank screen. Instructions were then presented on the left side of the display, remaining on the screen for 2 s (Fig. 4). Instructions consisted of a green arrow pointing upward (positive experience), a red arrow pointing downward (negative experience), or a blue circle (rest/neutral experience). Over the next 30 s, the screen remained unchanged, corresponding to the participant’s affective imagination period. Participants were previously instructed to actively imagine and maintain positive/negative affective experiences or to think nothing in particular during this period, based on the cues presented at the end of each trial. A new screen was then presented, and the participant was asked whether he or she was able to elicit the proper affective experience during the trial, and to again rate the intensity of the affective experienced on a scale ranging from 1–9. After this period, a blank screen was presented for a random duration between 2–4 s. Participants were instructed to blink during this period but not during the other phases of the experiment.

Trial order was randomized across participants, with a neutral trial between negative and positive trials. The active elicitation condition was the second block for all participants.

We used paired-Samples *t*-tests to evaluate potential differences in the intensity of affective experiences (positive/neutral/negative) and between conditions (active/passive), comparing the mean scores assigned by each participant in the positive and negative trials versus those assigned in the neutral trials. Bonferroni correction was applied based on the six comparisons performed (two elicitation conditions × three affective experiences).

### Acquisition of fNIRS data

**Figure 5:**
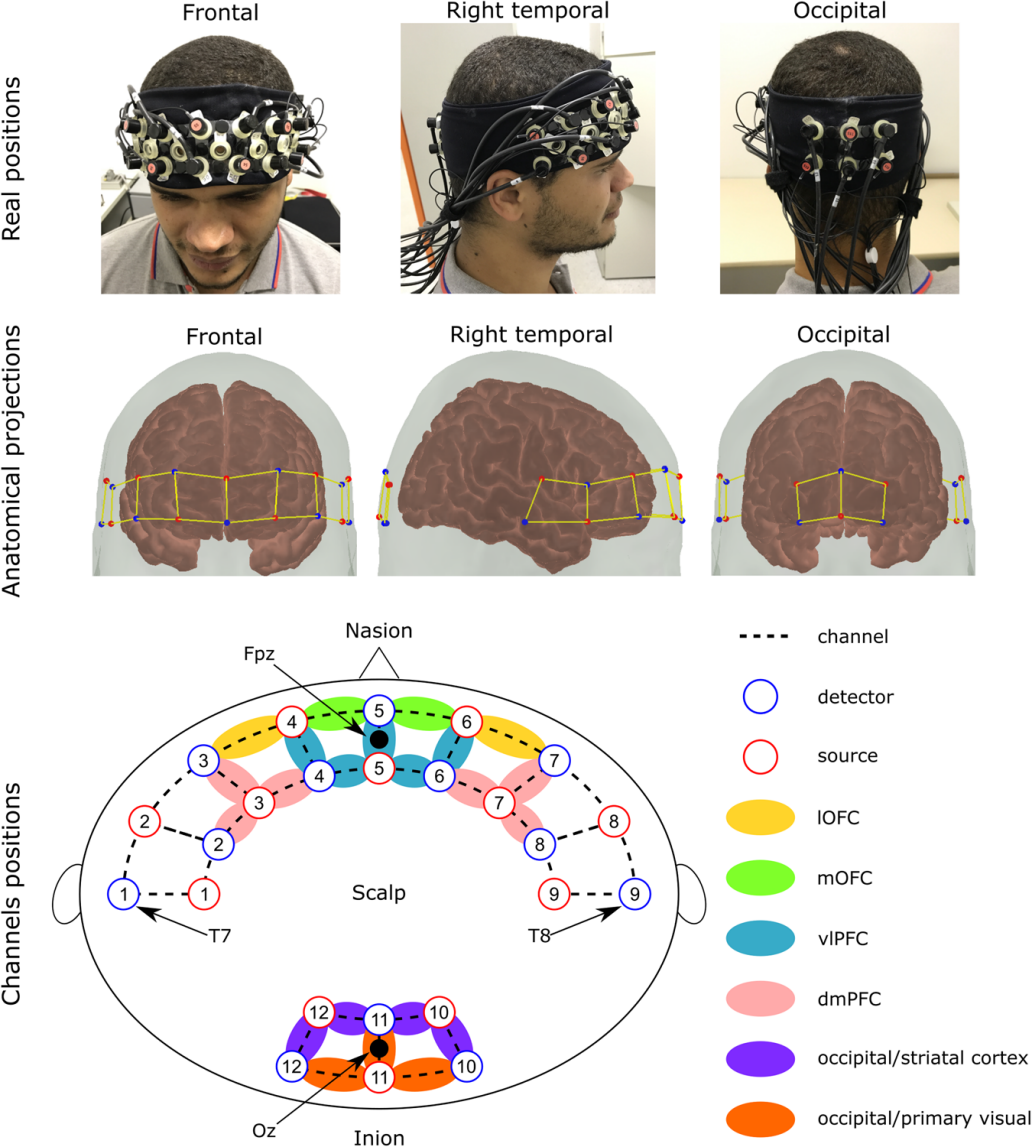
Real, anatomical, and schematic representations of channel configuration in three different perspectives. Red circles represent sources, while blue circles represent detectors. Dotted lines represent channels. The remaining colors represent the regions of interest for each channel: yellow for the lateral orbitofrontal cortex (lOFC), green for the medial orbitofrontal cortex (mOFC), blue for the ventrolateral prefrontal cortex (vlOFC), pink for the dorsomedial prefrontal cortex (dmPFC), purple for the occipital/striatal cortex, and orange for the occipital/primary visual cortex. The participant in this figure provided written informed consent for the publication of his photo in an online open-access publication.

An NIRScout System (NIRx Medical Technologies, LLC; Los Angeles, California) with a 32-channel array of optodes (12 light sources/emitters and 12 detectors) covering the prefrontal, temporal, and occipital areas was used to obtain fNIRS measurements (Fig. 5). Optodes were arranged in an elastic band, with nine sources and nine detectors positioned over the fronto-temporal regions, and three sources and three detectors over the occipital region. Four positions of the International 10–20 System were adopted as reference points during the setup: detectors 1 and 9 were positioned approximately over the T7 and T8 positions, respectively, while the Fpz and Oz positions were located in the center of channels 5–5 and 11–11, respectively. The source-receptor distance was 30 mm for contiguous optodes (which is considered the ideal source-detector separation for measuring cortical hemodynamics^47^), and two wavelengths were used (760 and 850 nm)^48^. Signals obtained from all 32 channels were measured at a sampling rate of 5.2083 Hz and recorded using NIRStar 14.0 software (NIRx Medical Technologies, LLC; Los Angeles, California).

### Data analysis

#### Preprocessing

Preprocessing was performed using Matlab (Mathworks, MA, USA) and the nirsLAB v2014.12 toolbox (NIRx Medical Technologies, LLC. Los Angeles, California). First, each participant’s raw data were digitally band-pass filtered using a linear-phase FIR filter (0.01–0.2 Hz). Then, each wavelength was baselined by the participant’s whole record (without segmentation), and the concentration curves for oxyhemoglobin and deoxyhemoglobin were calculated according to the Beer-Lambert law, with the differential pathlength factor (DPF) set to 7.25 and 6.38, respectively. Each concentration curve was then segmented into the 30 s of interest for each trial, for all studied affective experiences and elicitation conditions.

The mean concentration of oxyhemoglobin and deoxyhemoglobin for each segment and channel was calculated using a moving average of 2-s windows, with 50% overlap. Thus, a 20 × 64 matrix was created for each participant, with each line corresponding to one of the 20 trials (five trials of positive valence, five trials of negative valence, 10 trials of neutral valence). Columns represented the mean concentration of oxyhemoglobin and deoxyhemoglobin for the 32 total channels.

#### Affective experience decoding

We used a decoding approach^17^ to determine whether the extracted features were associated with affective experience. Linear discriminant analysis (LDA) is one of the simplest known classifiers and is commonly applied to classification problems. This method assumes that the two classes to be discriminated are normally distributed, with identical covariance^27^. Thus, the resultant prediction is a linear combination of all features based on their respective weights^49^. LDA is also advantageous for neuroimaging applications because it enables extraction of all discriminative information available in the sample, which may provide the necessary information for decoding neural phenomena^50^. In the present study, LDA was implemented using the default settings of the BCILAB toolbox^51^.

We first aimed to investigate whether different affective experiences could be decoded using fNIRS measures only. For each participant, an LDA classifier was applied using an intra-block leave-one-trial-out (LOTO) approach. In this method, the classifier was trained with *m* − 1 trials and tested on the remaining trials for each participant. This procedure was repeated *m* times, once for each trial in the dataset^27^. Pairwise comparisons of the three affective experiences (positive vs. neutral, negative vs. neutral, and positive vs. negative) were performed separately for the passive and active elicitation conditions. In order to identify which features (mean levels of oxyhemoglobin or deoxyhemoglobin) from each of the sampled cortical regions/channels were most relevant for the classification of the three affective experiences, we calculated the mean absolute weight values assigned by the classifier for each feature and for each LOTO iteration.

We then aimed to determine whether active and passive elicitation conditions are instantiated by the same cortical region, and whether they are associated with similar patterns of activation. For each participant, data from the passive elicitation condition were used as a training set, while data from the active elicitation condition were used as the test set for the LDA classifier, and vice versa. Comparisons were performed between valence experiences (positive vs. neutral, negative vs. neutral, and positive vs. negative) for each of these procedures.

Next, we investigated the extent to which active and passive elicitation conditions produced differentiable patterns of cortical hemodynamic signals. For each participant, data from the same affective experience in both active and passive conditions were examined using an inter-block LOTO approach. In this method, the classifier was trained with *m* − 1 trials and tested on the remaining trials for each participant. This procedure was repeated *m* times, once for each trial in the dataset^27^. Pairwise comparisons of the passive and active conditions were performed separately for the three affective experiences (positive vs. neutral, negative vs. neutral, and positive vs. negative).

We normalized both the training and test datasets as follows: First, we established the training (**T**) and test (**t**) sets for the current iteration; second, we calculated the mean (µ_T_) and standard-deviation (σ_T_) of the training set; third, µ_T_ was subtracted from both the training and test datasets, and the result was divided by σ_T_; fourth, the classifier was applied to the normalized values of **T** and **t**. We repeated this procedure for all iterations in all LOTO or cross-validation approaches. This normalization allows each set of features to have mean equals to zero and a variance of one. Finally, the performance of the classifier for each comparison was determined by calculating the decoding accuracy, which was given by the average number of trials correctly classified (i.e., the percentage of trials correctly classified from the first class plus the percentage of trials correctly classified from second class, divided by 2). The significance of classification accuracy was evaluated using a one-sample *t*-test against chance level (50%), and *p* values were Bonferroni corrected for multiple comparisons (12 tests). To evaluate the similarity between weight maps of interest (Figs 2a,b and 3a,b), we performed paired-samples t-tests comparing the inter-subject weights of each channel, in each pair of classification procedures (see Supplementary Material).

To explore potential confounders associated with the experimental procedure itself, we calculated the Spearman correlation coefficients between decoding accuracies and the variation of each measured mood variable before and after the experiment (VAMS questionnaire). The *p* values were again Bonferroni corrected for multiple comparisons (16 mood variables × 15 accuracy variables). Finally, the putative effects of gender were examined by comparing the decoding accuracies of men and women using a *t*-test for two independent samples (Bonferroni corrected for the 15 tests).

### Data availability

The datasets generated and/or analyzed during the current study are available from the corresponding author upon reasonable request.

## Electronic supplementary material

Supplementary Material
