# Supplementary material for: Predicting affective valence using cortical hemodynamic signals
Source: Sci Rep. 2018 Mar 29;8:5406. doi: 10.1038/s41598-018-23747-y (PMC5876393; doi:10.1038/s41598-018-23747-y)
Supplement: Supplementary file 1 — Supplementary Material [file 41598_2018_23747_MOESM1_ESM.pdf]

Predicting affective valence using cortical hemodynamic signals

Lucas R. Trambaiolli[1].

Claudinei E. Biazoli Jr[1].

André M. Cravo[1].

João R. Sato[1]

[1]Mathematics. Computation and Cognition Center. Universidade Federal do ABC - Rua Santa Adélia. 166 - Santo André - SP. 09210-170. Brazil.

Classification results using desoxyhemoglobin and oxyhemoglobin features separately

Table S1 - Means and standard deviations of decoding accuracy across subjects for each tested configuration. The first (Leave-one-trial-out intra-blocks) and third configurations (Leave-one-trial-out inter-blocks) were tested by the LOTO method. whereas the second configuration (Cross-validation inter-blocks) was trained with trials of one type of elicitation and tested with the other one.

			Desoxyhemoglobin features				***Oxyhemoglobin features***			
	**Comparison**		**Accuracy (%)**				**Accuracy (%)**			
	**Condition A**	**Condition B**	**(Conditions A+B)/2**	**Condition A**	**Condition B**	**p-value**	**(Conditions A+B)/2**	**Condition A**	**Condition B**	**p-value**
Leave-one-trial-out intra-blocks										
**Passive elicitation**	Positive	Neutral	**82.55±15.45**	**80.41±18.93**	**84.69±14.16**	**p<0.001**	**75.20±14.68**	**69.80±22.03**	**80.61±12.49**	**p<0.001**
	Negative	Neutral	**78.27±17.16**	**75.92±21.60**	**80.61±16.51**	**p<0.001**	**74.49±16.21**	**67.35±22.98**	**81.63±13.13**	**p<0.001**
	Positive	Negative	**59.39±15.47**	**60.00±18.26**	**58.78±19.75**	**p=0.001**	55.31±19.59	55.92±23.45	54.69±23.73	p=0.96
**Active elicitation**	Positive	Neutral	**88.78±14.27**	**86.12±18.35**	**91.43±11.73**	**p<0.001**	**84.90±13.86**	**81.63±18.18**	**88.16±12.53**	**p<0.001**
	Negative	Neutral	**88.06±17.26**	**84.49±25.58**	**91.63±11.61**	**p<0.001**	**87.76±14.07**	**86.53±17.98**	**88.98±12.46**	**p<0.001**
	Positive	Negative	45.71±20.62	43.27±27.19	48.16±20.38	p=1.00	49.18±20.70	51.02±22.38	47.35±25.07	p=1.00
Cross-validation inter-blocks										
**Passive x Active elicitation**	Positive	Neutral	55.00±23.56	49.39±29.75	60.61±22.31	p=1.00	55.10±20.98	46.94±28.74	63.27±24.44	p=1.00
	Negative	Neutral	54.29±23.78	44.49±29.51	64.08±26.21	p=1.00	52.96±21.43	43.67±32.06	62.25±23.65	p=1.00
	Positive	Negative	47.55±11.82	48.98±36.07	46.12±36.79	p=1.00	51.63±16.63	57.14±32.91	46.12±34.21	p=1.00
**Active x Passive elicitation**	Positive	Neutral	49.29±17.11	43.27±24.95	55.31±20.42	p=1.00	53.06±17.26	48.16±27.96	57.96±21.41	p=1.00
	Negative	Neutral	51.33±18.17	48.16±25.79	54.49±18.38	p=1.00	54.49±17.71	49.39±23.49	59.59±23.00	p=1.00
	Positive	Negative	50.41±12.58	43.67±32.06	57.14±33.42	p=1.00	49.39±15.60	46.12±34.21	52.65±27.90	p=1.00
Leave-one-trial-out inter-blocks										
**Neutral**			**80.41±13.42**	**82.25±14.76**	**78.57±15.14**	**p<0.001**	**79.18±13.86**	**81.63±13.90**	**76.74±17.61**	**p<0.001**
**Negative**	Active elicitation	Passive elicitation	**73.06±17.70**	**73.06±21.43**	**73.06±22.93**	**p<0.001**	**71.43±16.20**	**73.47±20.57**	**69.39±20.45**	**p<0.001**
**Positive**			**72.86±19.15**	**73.47±23.59**	**72.25±21.14**	**p<0.001**	**71.43±16.96**	**72.25±17.23**	**70.61±21.64**	**p<0.001**

Similarity of the deoxyhemoglobin weight maps

We used paired-samples t-tests to evaluate the similarity of weights assigned into the same channel during different classification procedures (for example, comparing inter-subject weights of channel 1-1 during positive vs. neutral classification reported in Figure 2A and during negative vs. neutral classification reported in Figure 2B). Figure S1 illustrates the comparisons performed here.

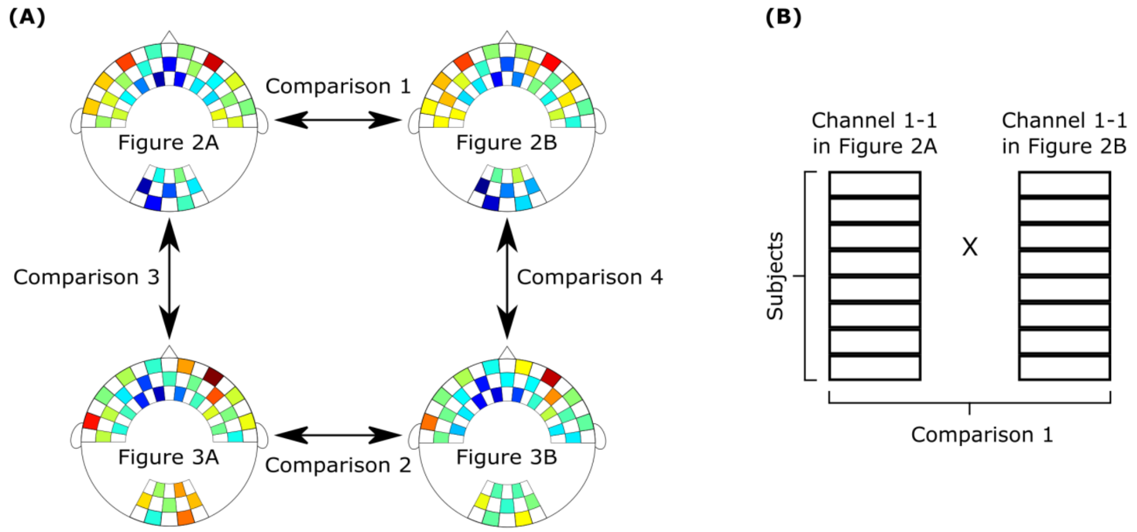

Figure S1 – (A) Definition of the pairs of maps compared, and (B) an example of a comparison setup.

Table S2 shows the respective p-values for each channel, in each comparison. Using 5% of confidence and Bonferroni correction for multiple comparisons (32 channels x 4 comparisons), p-values should be smaller than 0,00039 to reach significance.

Table S2 – Resulting p-values for each channel, in each comparison illustrated in Figure S1. The significant channel is highlighted in bold.

**Channel**	**Comparison 1**	**Comparison 2**	**Comparison 3**	**Comparison 4**
1 - 1	0.2783	0.5215	0.6015	0.1856
1 - 2	0.3957	0.2606	0.3948	0.0745
2 - 1	0.3496	0.6327	0.3431	0.6350
2 - 2	0.4675	0.2239	0.4004	0.0472
2 - 3	0.9941	0.4281	0.1649	0.0375
3 - 2	0.1050	0.7730	0.6144	0.9929
3 - 3	0.3684	0.9606	0.1457	0.0699
3 - 4	0.3032	0.2013	0.6146	0.1634
4 - 3	0.7906	0.9581	0.0932	0.0480
4 - 4	0.8124	0.6309	0.3416	0.3094
4 - 5	0.7611	0.8409	0.2539	0.5108
5 - 4	0.8303	0.5460	0.7025	0.9150
5 - 5	0.2791	0.2117	0.0032	0.0067
5 - 6	0.7232	0.9189	0.7800	0.5818
6 - 5	0.4285	0.5507	0.1455	0.4828
6 - 6	0.5782	0.2080	0.4938	0.0975
6 - 7	0.4264	0.3397	0.9670	0.8071
7 - 6	0.6364	0.2883	0.5324	0.7967
7 - 7	0.4599	0.4263	0.0045	0.0506
7 - 8	0.3439	0.7858	0.1326	0.1861
8 - 7	0.6517	0.2295	0.9198	0.1635
8 - 8	0.6236	0.0673	0.6598	0.0831
8 - 9	0.0249	0.5529	0.0969	0.8114
9 - 8	0.2105	0.4671	0.3022	0.1786
9 - 9	0.4511	0.0193	0.0640	0.9068
10 - 10	0.0162	0.4788	0.0525	0.4637
10 - 11	0.0607	0.8937	0.7946	0.5117
11 - 10	0.5976	0.3413	0.4979	0.2943
11 - 11	0.3467	0.7507	0.1167	0.2473
11 - 12	0.1126	0.1219	0.5778	0.0104
12 - 11	0.3308	0.9401	0.7280	0.4478
12 - 12	0.9545	0.4932	0.0134	**0.0002**
